# Supplementary material for: Negative attitudes about facemasks during the COVID-19 pandemic: The dual importance of perceived ineffectiveness and psychological reactance
Source: PLoS One. 2021 Feb 17;16(2):e0246317. doi: 10.1371/journal.pone.0246317 (PMC7888611; doi:10.1371/journal.pone.0246317)
Supplement: S1 Appendix — (DOCX) [file pone.0246317.s001.docx]

**S1 Appendix. Mask Attitudes Study**

| **Edge weights for Figure 1** | |  |  | **Edge weights for Figure 3** | | |
| --- | --- | --- | --- | --- | --- | --- |
| **Edge** | **Weight** | **p** |  | **Edge** | **Weight** | **p** |
| SIL-UGL | 0.60 | <.001 |  | UGL-SIL | 0.65 | <.001 |
| HEA-BRE | 0.59 | <.001 |  | BRE-HEA | 0.59 | <.001 |
| UNE-UNT | 0.47 | <.001 |  | HAS-HAB | 0.40 | <.001 |
| FAC-INE | 0.42 | <.001 |  | FAC-INE | 0.40 | <.001 |
| HAB-HAS | 0.40 | <.001 |  | EXA-ROB | 0.28 | <.001 |
| INE-SEC | 0.40 | <.001 |  | EXA-DIS | 0.25 | <.001 |
| PR-INE | 0.17 | <.001 |  | PR-EXA | 0.19 | <.001 |
| PR-HAB | 0.16 | <.001 |  | FAC-SEC | 0.16 | <.001 |
| PR-HAS | 0.16 | <.001 |  | HAS-PR | 0.15 | <.001 |
| FAC-SEC | 0.15 | <.001 |  | HAB-PR | 0.15 | <.001 |
| UNT-UGL | 0.14 | <.001 |  | EXA-POL | 0.15 | <.001 |
| UNE-PR | 0.12 | <.001 |  | UGL-UNT | 0.14 | <.001 |
| SIL-PR | 0.12 | <.001 |  | EXA-VAX | 0.14 | <.001 |
| UNT-SIL | 0.09 | <.001 |  | INE-EXA | 0.13 | <.001 |
| BRE-HAS | 0.09 | <.001 |  | UNE-PR | 0.12 | <.001 |
| UNE-UGL | 0.08 | <.001 |  | PR-INE | 0.12 | <.001 |
| UNT-PR | 0.08 | <.001 |  | SIL-PR | 0.11 | <.001 |
| UNT-FAC | 0.08 | <.001 |  | ROB-DIS | 0.11 | <.001 |
| HEA-SEC | 0.08 | <.001 |  | SIL-UNT | 0.09 | <.001 |
| HAS-FAC | 0.08 | <.001 |  | HAS-BRE | 0.09 | <.001 |
| UNT-HAS | 0.07 | <.005 |  | UGL-UNE | 0.08 | <.001 |
| UGL-HAS | 0.07 | <.005 |  | UNT-PR | 0.08 | <.001 |
| HEA-FAC | 0.07 | <.005 |  | UNT-FAC | 0.08 | <.001 |
| HAS-INE | 0.07 | <.005 |  | HAS-FAC | 0.08 | <.001 |
| UNE-SIL | 0.06 | <.01 |  | HEA-SEC | 0.08 | <.001 |
| SIL-HAB | 0.06 | <.01 |  | FAC-VAX | 0.08 | <.001 |
| UGL-PR | 0.06 | <.01 |  | INE-DIS | 0.08 | <.001 |
| HEA-HAS | 0.06 | <.01 |  | UGL-HAS | 0.07 | <.005 |
| BRE-HAB | 0.06 | <.01 |  | UNT-HAS | 0.07 | <.005 |
| BRE-FAC | 0.06 | <.01 |  | PR-POL | 0.07 | <.005 |
| HAB-FAC | 0.06 | <.01 |  | INE-VAX | 0.07 | <.005 |
|  |  |  |  | UGL-PR | 0.06 | <.01 |
|  |  |  |  | SIL-HAB | 0.06 | <.01 |
|  |  |  |  | SIL-UNE | 0.06 | <.01 |
|  |  |  |  | HAS-HEA | 0.06 | <.01 |
|  |  |  |  | HAS-INE | 0.06 | <.01 |
|  |  |  |  | BRE-FAC | 0.06 | <.01 |
|  |  |  |  | HAB-FAC | 0.06 | <.01 |
|  |  |  |  | HAB-BRE | 0.06 | <.01 |

Note: *BRE:* It is difficult to breathe when wearing a facemask. *FAC:* Facemasks are unsafe because they force you to touch your face. *HAB:* It is hard to develop the habit of wearing a facemask. *HAS:* Wearing a facemask is too much of a hassle. *HEA:* Facemasks cause me to overheat. *INE:* Facemasks are ineffective. *PR:* I do not like feeling forced to wear a facemask. *SEC:* Facemasks provide a false sense of security. *SIL:* Facemasks look silly. *UGL:* Facemasks look ugly or weird. *UNE:* Facemasks make other people feel uneasy. *UNT:* Facemasks make people look untrustworthy.

**Correlations with demographic variables**

|  | Age | Female  gender | College  education | Unemployed | Country  (1=Canada, 2=US) | Ethnic  minority |
| --- | --- | --- | --- | --- | --- | --- |
| Wear a mask  because of COVID-19  concerns? (1=yes, 0=no) | -.03 | -.01 | .02 | -.02 | .13*** | .07*** |
| BRE | .00 | .04 | .00 | -.01 | .04 | -.02 |
| HEA | -.02 | .03 | .02 | .03 | -.02 | -.05 |
| SEC | .00 | .01 | .03 | .01 | .01 | -.03 |
| INE | -.02 | .00 | .02 | .02 | .02 | -.02 |
| FAC | -.03 | .01 | .04 | .04 | -.02 | -.04 |
| HAS | -.01 | .00 | -.01 | .04 | -.06 | -.02 |
| HAB | -.01 | -.02 | .01 | .06 | -.08*** | -.07** |
| SIL | .01 | -.02 | .01 | .04 | .02 | -.06* |
| UGL | .01 | -.02 | .01 | .04 | .03 | -.05 |
| UNT | .00 | -.04 | .03 | .04 | .05 | -.02 |
| UNE | .03 | -.05 | .01 | .02 | .02 | -.04 |
| PR | .01 | -.05 | -.01 | .02 | .03 | -.06** |

Note: *BRE:* It is difficult to breathe when wearing a facemask. *FAC:* Facemasks are unsafe because they force you to touch your face. *HAB:* It is hard to develop the habit of wearing a facemask. *HAS:* Wearing a facemask is too much of a hassle. *HEA:* Facemasks cause me to overheat. *INE:* Facemasks are ineffective. *PR:* I do not like feeling forced to wear a facemask. *SEC:* Facemasks provide a false sense of security. *SIL:* Facemasks look silly. *UGL:* Facemasks look ugly or weird. *UNE:* Facemasks make other people feel uneasy. *UNT:* Facemasks make people look untrustworthy. *p<.01, **p<.005, ***p<.001

**Correlations among variables used in the network analyses**

|  | BRE | HEA | SEC | INE | FAC | HAS | HAB | SIL | UGL | UNT | UNE | PR | POL | VAX | DIS | EXA |
| --- | --- | --- | --- | --- | --- | --- | --- | --- | --- | --- | --- | --- | --- | --- | --- | --- |
| HEA | .74 |  |  |  |  |  |  |  |  |  |  |  |  |  |  |  |
| SEC | .43 | .45 |  |  |  |  |  |  |  |  |  |  |  |  |  |  |
| INE | .45 | .44 | .75 |  |  |  |  |  |  |  |  |  |  |  |  |  |
| FAC | .47 | .45 | .67 | .80 |  |  |  |  |  |  |  |  |  |  |  |  |
| HAS | .49 | .46 | .53 | .62 | .61 |  |  |  |  |  |  |  |  |  |  |  |
| HAB | .44 | .41 | .47 | .53 | .55 | .72 |  |  |  |  |  |  |  |  |  |  |
| SIL | .37 | .38 | .49 | .53 | .54 | .61 | .56 |  |  |  |  |  |  |  |  |  |
| UGL | .38 | .39 | .48 | .53 | .54 | .61 | .55 | .91 |  |  |  |  |  |  |  |  |
| UNT | .38 | .37 | .49 | .58 | .58 | .60 | .53 | .69 | .71 |  |  |  |  |  |  |  |
| UNE | .35 | .36 | .47 | .51 | .52 | .55 | .50 | .65 | .66 | .78 |  |  |  |  |  |  |
| PR | .43 | .42 | .55 | .66 | .61 | .68 | .63 | .67 | .65 | .64 | .61 |  |  |  |  |  |
| POL | .16 | .10 | .27 | .30 | .25 | .23 | .19 | .24 | .22 | .23 | .21 | .32 |  |  |  |  |
| VAX | .25 | .22 | .33 | .43 | .42 | .33 | .26 | .27 | .27 | .34 | .30 | .33 | .20 |  |  |  |
| DIS | .20 | .17 | .32 | .46 | .40 | .38 | .31 | .34 | .34 | .39 | .31 | .40 | .18 | .34 |  |  |
| EXA | .30 | .27 | .47 | .62 | .54 | .49 | .44 | .47 | .47 | .49 | .41 | .62 | .37 | .43 | .57 |  |
| ROB | .09 | .10 | .25 | .32 | .30 | .29 | .25 | .31 | .31 | .28 | .25 | .36 | .17 | .26 | .40 | .53 |

Note: *BRE:* It is difficult to breathe when wearing a facemask. *FAC:* Facemasks are unsafe because they force you to touch your face. *HAB:* It is hard to develop the habit of wearing a facemask. *HAS:* Wearing a facemask is too much of a hassle. *HEA:* Facemasks cause me to overheat. *INE:* Facemasks are ineffective. *PR:* I do not like feeling forced to wear a facemask. *SEC:* Facemasks provide a false sense of security. *SIL:* Facemasks look silly. *UGL:* Facemasks look ugly or weird. *UNE:* Facemasks make other people feel uneasy. *UNT:* Facemasks make people look untrustworthy. All ps<.001.
